# On-Chip Modification of Titanium Electrothermal Characteristics by Joule Heating: Application to Terahertz Microbolometer

**DOI:** 10.3390/nano14020225

**Published:** 2024-01-19

**Authors:** Durgadevi Elamaran, Ko Akiba, Hiroaki Satoh, Amit Banerjee, Norihisa Hiromoto, Hiroshi Inokawa

**Affiliations:** 1 Graduate School of Science and Technology, Shizuoka University, Hamamatsu 432-8011, Japan; hiromoto.norihisa@shizuoka.ac.jp; 2 Graduate School of Integrated Science and Technology, Shizuoka University, Hamamatsu 432-8561, Japan; akiba.ko.17@shizuoka.ac.jp (K.A.); satoh.hiroaki@shizuoka.ac.jp (H.S.); 3 Research Institute of Electronics, Shizuoka University, Hamamatsu 432-8011, Japan

**Keywords:** titanium, titanium oxide, Joule heating, temperature coefficient of resistance (TCR), microbolometer, responsivity, noise equivalent power (NEP), transmission electron microscopy (TEM), energy dispersive X-ray analysis (EDX)

## Abstract

This study demonstrates the conversion of metallic titanium (Ti) to titanium oxide just by conducting electrical current through Ti thin film in vacuum and increasing the temperature by Joule heating. This led to the improvement of electrical and thermal properties of a microbolometer. A microbolometer with an integrated Ti thermistor and heater width of 2.7 µm and a length of 50 µm was fabricated for the current study. Constant-voltage stresses were applied to the thermistor wire to observe the effect of the Joule heating on its properties. Thermistor resistance ~14 times the initial resistance was observed owing to the heating. A negative large temperature coefficient of resistance (TCR) of −0.32%/K was also observed owing to the treatment, leading to an improved responsivity of ~4.5 times from devices with untreated Ti thermistors. However, this does not improve the noise equivalent power (NEP), due to the increased flicker noise. Microstructural analyses with transmission electron microscopy (TEM), transmission electron diffraction (TED) and energy dispersive X-ray (EDX) confirm the formation of a titanium oxide (TiO_x_) semiconducting phase on the Ti phase (~85% purity) deposited initially, further to the heating. Formation of TiO_x_ during annealing could minimize the narrow width effect, which we reported previously in thin metal wires, leading to enhancement of responsivity.

## 1. Introduction

Titanium (Ti) is widely used in silicon (Si) integrated circuits as an adhesion layer between Si and metals and dielectrics and metals [1], and on-chip improvement or modifications of their characteristics are of great interest. For example, on-chip controlled oxidation may lead to a new type of photocatalyst, dye-sensitized solar cell, batteries, etc. [2]. Ti also features high electrical and thermal resistances, which make it attractive as a thermistor/heater material for microbolometers, a kind of thermal radiation detector, for infrared and terahertz (THz) waves. The frequency region spanning 300 gigahertz (GHz) to 3 THz has a wide range of applications in various fields such as security screening, medical imaging and industrial quality control [1,2,3,4,5]. THz radiation exhibits photon energy from 1.2 to 12 meV, which may be related to the blackbody radiation peak energy range around ~10 K. Hence, any object with a temperature higher than 10 K emits THz radiation [6]. The unique property of this radiation range is that it is non-ionizing because of its low photon energy, and hence it is not harmful for living tissues or DNA. This also enables higher-resolution images than with microwaves and millimeter waves, with high penetration capability [7,8], and hence it can be used for medical screening, diagnosis and imaging, material identification, illicit drug detection, food testing, security screening, etc. [1,9,10,11,12,13,14,15]. Due to its many interesting properties, THz radiation comes with the possibility of the realization and commercialization of numerous room-temperature, sensitive detectors. However, while many devices are commercialized for microwave and infrared ranges, those for THz waves are lacking. There is an urgent need for the development of room-temperature, highly sensitive THz detectors. Currently, the major categories of THz detectors are photon detectors, thermal detectors and electronic receivers. Photon detectors are sensitive and already commercialized widely for laboratory use, but they operate at low frequencies (small photon energy), requiring cryogenic cooling for noise reduction. Electronic receivers based on Schottky barrier diodes, field-effect transistors (FETs) and hetero bipolar transistors (HBTs) show lower sensitivity in higher-frequency ranges. However, this has been gradually resolved with the advancement in materials engineering and microfabrication technology. On the other hand, today thermal detectors are reliable, cost-effective and capable of room-temperature operation, making them suitable for a wide range of applications. Thermal detectors offer moderate sensitivity and video-rate speed, with internal thermal conductance of ~10^−8^ W/K and heat capacity of less than 10^−9^ J/K [16]. Thermal detectors rely on changes in material properties consistent with the temperature rise caused by the input radiated signal [17,18]. In such a way, thermal detectors, represented by bolometers, in which the temperature rise is detected as a change in electrical resistance, are capable of THz sensing and imaging [19]. A bolometer generally consists of an absorber/heater and a temperature-sensing resistor (thermistor), with the size of the absorber sufficiently larger than the minimum spot size of the incident light limited by diffraction. Hence, it needs to be larger than λ/n_e_ for the wavelength of λ and effective refractive index of n_e_ [20]. However, for THz waves with longer wavelengths, the absorber size becomes too large to sustain the thermal insulating structure and to realize low heat capacity and hence an antenna-coupled bolometer design is more viable. Antenna-coupled microbolometers fabricated by MEMS technology have been extensively studied in the past three decades [20,21,22,23], where the radiation is absorbed by an antenna and converted to heat by a load resistor (heater), then the corresponding input radiation changes the properties of the thermistor material. Antenna-coupled microbolometers with a separate heater and thermistor have also been studied extensively by our group to build the foundation for the current study [24,25,26,27]. The design and development of efficient room-temperature microbolometers with prospects for on-chip fabrication, aided by current advancements in materials engineering microfabrication technologies, have enormous possibilities leading to mainstream applications in terahertz spectroscopic studies and imaging. THz wavelengths (100 μm to 1 mm) yield extremely high-resolution imaging; move vast amounts of data quickly; and are non-ionizing (do not trigger harmful chemical reactions) for human tissue on extended exposure unlike X-rays. Diagnostics of defects in materials and foods and investigating chemical compositions are also possible by detection of weak inter-molecule coupling and molecular networks [10,11,28,29,30,31,32]. Further, there have been some systematic breakthroughs in the past decade in the development of room-temperature detectors, high-power sources and real-time imaging in THz [33,34,35,36].

Based on our familiarity in this field from previous extensive studies on the design and development of Ti on-chip microbolometers [24,25,26,27,37,38,39,40], the current report is focused on the on-chip modification of Ti electrothermal characteristics after fabrication by Joule heating, aiming at a terahertz microbolometer as a possible application. Our previous understanding on the importance of tuning of material properties like narrow width effect, grain size, TCR and resistivity in feature miniaturization for enhanced device performance, responsivity and NEP has been useful here. The on-chip integrable microbolometer device array made in our previous studies with nanometer-width Ti meander thermistors does come with scope for further miniaturization, suited to standard semiconductor fabrication technology. These devices may not be adequate for thermal imaging with acceptable integration times but can be employed in imaging systems that utilize an active THz source. Comparative analyses of these devices with other materials (Vox, VO_2_, Ti, SiGe, etc. as sensing material) and devices (MOSFET, PN junction diode and resistor-based sensors) have also been reported previously [27,38]. While we have previously established that the narrow width effect is detrimental to superior device performance, and it is understood that the meander shape helps the enhanced responsivity but does not improve NEP, here we have proposed a method in nanomaterial processing to further enhance the device performance by on-chip modification of Ti thermistors after fabrication by a feasible Joule heating and highlighted the possible issues that may be encountered by technologists. Hence, even though the microbolometer performance is not so tempting in these devices, the nanomaterial-processing point of view is unique and, to the best of our knowledge, has not been addressed in any other literature with such details for on-chip terahertz microbolometers and other Ti-based devices. Furthermore, we have reported comprehensive review studies on the possibility for biomedical spectroscopy and medical applications of terahertz waves [39] and proposed image-processing techniques for possible use in medical imaging [40].

Beyond these, a narrow band microbolometer sensor (NBMS) with a novel diffractive lens for spectroscopic microscopy was reported [41] for non-neoplastic and neoplastic human colon tissues using THz imaging for the first time, which demonstrated the new potential of compact imaging systems in the fields of spectroscopic analysis of materials and medical diagnostics. Further to medical diagnostics, the possibilities of terahertz nanoimaging for solar cells and thin semiconductors with on-chip Ti microbolometers is also exciting and currently under investigation. It is interesting to note that solar cell degradation and the influence of various environmental factors on their performance remain important challenges. THz can penetrate many non-conductive materials, including organic and inorganic thin films used in solar cell fabrication [42] and helps predict solar cell performance with terahertz–microwave spectroscopy that can infer critical parameters such as carrier lifetime and mobility and even determine potential power conversion efficiency. This predictive capability is invaluable as it allows for early detection of degradation and performance loss, enabling timely maintenance or replacement of solar cells before they significantly impact overall energy production [43,44]. Also, assessing the degradation of polycrystalline silicon solar cells, which are widely used in commercial solar panels, with a laser terahertz emission microscope (LTEM) presents an alternative approach to analyzing these cells non-destructively. The LTEM operates by exciting the solar cell with a pulsed laser, causing it to emit THz radiation. This emitted THz radiation is then analyzed to extract valuable information about the material’s properties and performance. Comparing the LTEM with conventional methods, such as electroluminescence and infrared imaging, reveals several advantages. The LTEM provides detailed information on charge carrier mobility, trap states and even sub-surface defects, offering a more comprehensive understanding of the solar cell’s health [45]. Further optical and THz reflectance investigations of organic solar cells have emerged as valuable techniques to probe the interfacial properties, crystallinity and charge carrier dynamics within cells. THz imaging provides the ability to study these cells with higher spatial resolution and sensitivity. It allows researchers to detect sub-surface defects, monitor morphological changes and evaluate the impact of degradation over time. Moreover, the non-invasive nature of THz imaging ensures that the structural integrity of the organic solar cells remains intact throughout the assessment process [46]. Furthermore, the scanning laser THz imaging system represents a pioneering approach to inspecting solar cells at the microscale level by combining the advantages of THz radiation with precise laser scanning technology, enabling comprehensive imaging of solar cell properties. The system scans the solar cell surface with a focused THz beam, generating high-resolution images of material properties and degradation patterns, identifying regions of interest, such as cracks, defects or variations in material composition, which are crucial in understanding the mechanisms behind solar cell performance degradation. The real-time imaging capability facilitates rapid data acquisition and improves the efficiency of solar cell evaluation [47]. THz imaging technology has proven to be a powerful tool for inspecting the degradation and conditions of solar cells due to its non-destructive nature, high spatial resolution and ability to predict performance, making it an indispensable asset in the renewable energy industry. As research in this field continues to evolve, THz imaging is expected to play an increasingly significant role in enhancing the efficiency and lifespan of solar cell technologies, contributing to a greener and more sustainable future.

While our microbolometer device arrays have shown reasonable responsivity with improved noise-equivalent power (NEP) for imaging, with potential for application in imaging systems with active THz sources with acceptable integration times, further development is underway. Further, we have also recently reported a chip-integrable solid-state THz source with an AlGaN/GaN-based Schottky barrier lateral HEM-ATT that can deliver a notable ~300 mW, operating at a 1.0 THz frequency [48]. The microbolometer and source may be suitable for development of compact solid-state spectroscopy systems.

The thermistor material for the microbolometer has a significant influence on the detector’s sensitivity. Specifically, it is important to have a material with appropriate resistance and TCR [49], since the responsivity (*R*_v_) is proportional to them. In comparison with high-TCR materials like vanadium oxide (VO_x_) and amorphous silicon (a-Si), pure metals have a relatively small TCR but show reduced noise consisting mainly of thermal and shot noises that are not correlated to the TCR [25], and hence the performance of the bolometer has a direct benefit from the improved TCR. In the current work, Ti is selected as a thermistor–heater material, considering its low thermal and electrical conductivities, immunity to electromigration and low flicker noise [37,49,50,51]. However, the TCR of thin and narrow Ti metallic wires is largely affected by the presence of defects or grain boundaries [25], leading to the narrow width effect in thin metal wires reported by us previously. Here, the optimization of the narrow width effect in nanoscale titanium thermistors further to controlled annealing by Joule heating has been explored. Among various methods of annealing, such as furnace annealing, laser annealing and Joule heating by direct current through the resistor, the latter is selected considering the possibility of multiple trials in a single chip and advantage of in situ monitoring of the annealing process.

In order to analyze the effects of annealing, a Ti straight wire integrated thermistor and heater were fabricated with a width of 2.7 µm, length of 50 µm and a thickness of 77 nm. Deposited Ti film has relatively higher resistivity than that of the bulk material due to its polycrystalline structure. The desired outcomes of this controlled annealing include:

(1) Crystalline defects, such as vacancy, interstitial, dislocation, etc., to be reduced, resulting in lower resistivity and TCR closer to that of bulk material.
(2) Grain size increase, also resulting in lower resistivity and TCR closer to that of bulk material. This may alleviate the narrow width effect caused by the small grain size.
(3) Phase separation in the case of multi-element material, i.e., where impurities are included unintentionally.

This paper includes the comprehensive details of device design, experimental methods, measurement techniques and device performance analysis. Here, the electrical and thermal properties of the Ti thermistor were modified by the process of Joule heating. Microstructural analysis was carried out with transmission electron microscopy and energy dispersive X-ray (TEM/EDX) analysis of the Ti thermistor before and after Joule heating to correlate the electrical and device results. Further to our reports on chip-integrable uncooled terahertz microbolometer arrays, compatible with medium-scale semiconductor device fabrication processes, the current study explores performance improvement by minimization of narrow width effect in thin titanium thermistors with annealing.

## 2. Materials and Methods

Oxidized silicon substrate with oxide thickness of 400 nm was used for the fabrication of Ti microbolometers. Ti wire was formed by patterning of electron-beam (EB) evaporated Ti thin film using laser lithography and liftoff. After that, the deep cavity for thermal isolation was formed by CHF_3_ RIE and SF_6_ plasma etching. Details of the fabrication process have been elaborated on in our previous publications [24,25,26,27,37,38].

**Figure 1:**
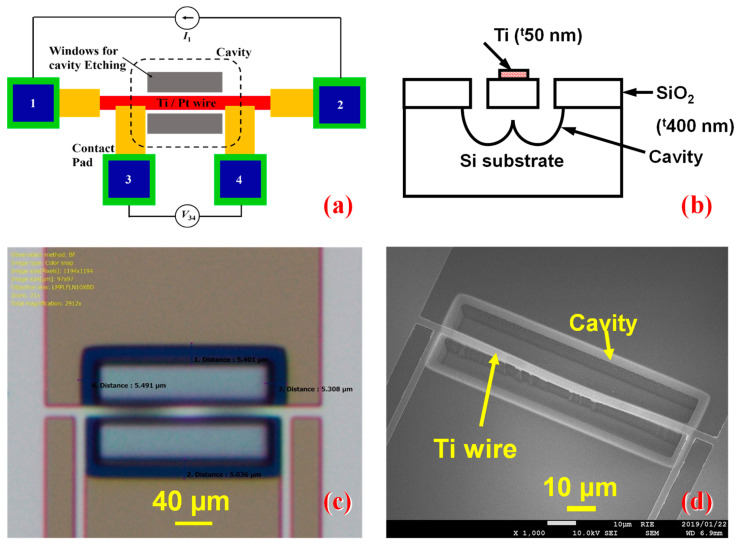
(**a**) Planar and (**b**) cross-sectional views of the suspended Ti thermistor. (**c**) Optical and (**d**) SEM images of the fabricated thermistor.

**Figure 2:**
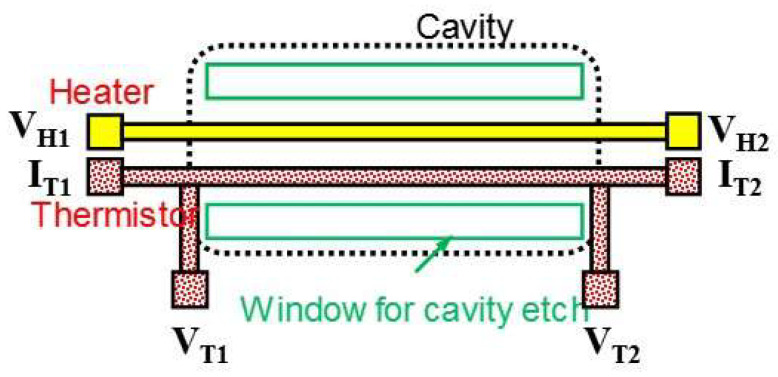
Planar view of the microbolometer. An antenna shall be connected to the heater to receive electromagnetic waves and to raise the temperature of the thermistor.

To verify the effect of Joule heating, initially, Ti thermistor wires without the heater were fabricated as shown in Figure 1a–d. Figure 1a,b represent the schematic and cross-sectional views of the suspended Ti thermistor and Figure 1c,d represent the corresponding optical and SEM images, which show the formation of Ti thermistor wire with a length of 50 µm and a width of 2 µm. A deep cavity was formed to provide better thermal isolation from the substrate. Subsequently, a similar kind of thermistor with a heater and cavity as that in a suspended wire thermistor (heater/thermistor length: 50 µm and heater/thermistor width: 2 µm) was fabricated to confirm the effect of Joule heating on microbolometers. The width and thickness of the fabricated structure measured by FE-SEM and step profiler are 2.7 µm and 77 nm, respectively. A schematic view of the fabricated microbolometer is shown in Figure 2, in which the heater and thermistor were fabricated in parallel with identical device dimensions. During the responsivity (*R*_v_) analysis, the thermistor’s current terminals *I*_T1_ and *I*_T2_ of the bolometer were used for applying input bias current, whereas the output voltage was recorded between the thermistor’s voltage terminals *V*_T1_ and *V*_T2_. Heater input power was excited through the voltage terminals *V*_H1_ and *V*_H2_.

Electrical analyses for the fabricated bolometer were carried out using a Nagase Techno-Engineering Grail 21-205-6-LV-R (Grail, Menlo Park, CA, USA) temperature-controlled vacuum prober equipped with a Keysight 4156C (Agilent Technologies, Santa Clara, CA, USA) precision semiconductor parameter analyzer. The Ti heater and thermistor TCRs were tested with temperature ranges from 260 to 300 K from the slope of resistance vs. temperature. A Cryogenic Control Systems Inc. (Rancho Santa Fe, CA, USA) Cryocon 32 temperature controller installed inside the prober was used to monitor the sample temperature with an accuracy of ±0.2 K. Responsivity was calculated by applying AC input power to the heater with a frequency of 10 Hz. The output voltage from the thermistor was recorded using a Signal Recovery 7270 (Ametek, Newark, DE, USA) lock-in amplifier under low bias current such that it could provide a reasonable temperature rise at the center wire. The thermistor’s noise characteristics were evaluated using a low-temperature prober with the FFT dynamic signal analyzer Keysight 35670A (Keysight, Santa Rosa, CA, USA). The noise signal was amplified with the DL Instruments 1201 (OEM, Tokyo, Japan) low-noise voltage preamplifier. The noise spectrum was measured from 1 Hz to 100 kHz over the frequency range.

## 3. Results and Discussion

### 3.1. Comparative Study: Before and after Joule Heating

**Figure 3:**
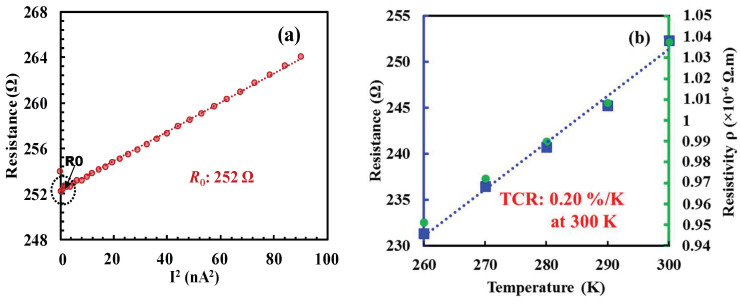
Electrical characteristics of the suspended wire thermistor before Joule heating. (**a**) Resistance vs. squared current, and (**b**) zero-biased resistance *R*_0_ and resistivity vs. temperature.

**Figure 4:**
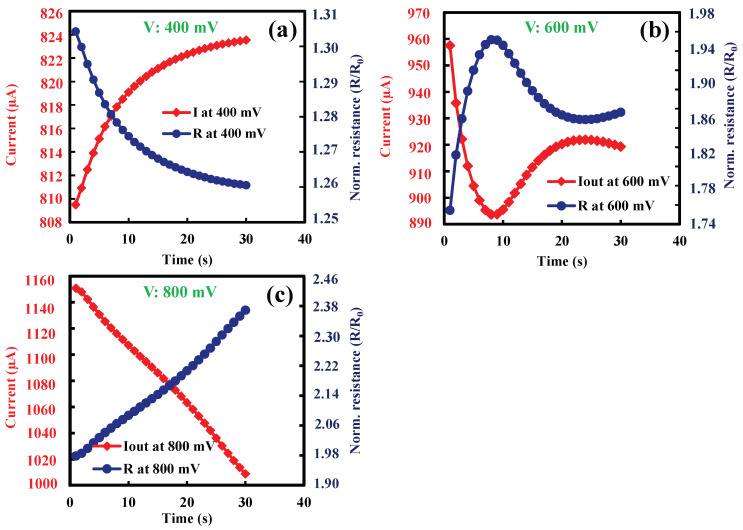
Current and normalized resistance (*R*/*R*_0_) during the Joule heating with the steps of (**a**) 400 mV, (**b**) 600 mV and (**c**) 800 mV. The room-temperature resistance *R*_0_ = 252 Ω (Figure 3a).

Initially, the effect of Joule heating was verified on suspended wire thermistors without a heater. Resistance, resistivity and TCR of the suspended wire thermistor were measured before employing Joule heating. Applying an electrical current across the wire from one end to the other and recording the voltage difference at room temperature enable the determination of resistance by dividing the recorded voltage difference by the applied current. The TCR was obtained by measuring resistances at different temperatures. Figure 3a illustrates the relationship between the resistance of the suspended wire thermistor and the square of the input current, while Figure 3b depicts the extracted TCR. Joule heating was employed on thermistor wire by applying a large constant voltage instead of current through the wire, as a large current may lead to thermal runaway and hence device breakdown. To avoid the breakage of the device, constant voltage was applied for 30 s and increased step by step. The change in resistance and current during the Joule heating for different steps of constant voltage such as 400 mV, 600 mV and 800 mV is shown in Figure 4. The normalized resistance increases with the constant step voltages as a result of material property change.

**Figure 5:**
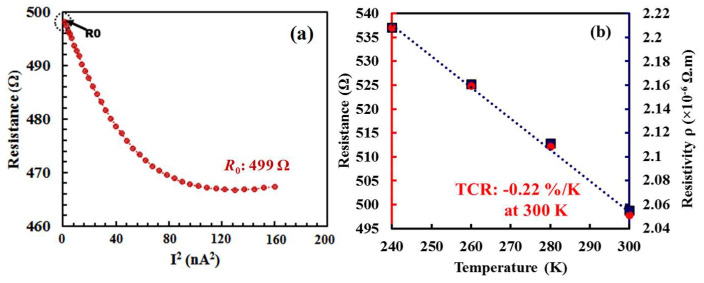
Electrical characteristics of the suspended wire thermistor after Joule heating. (**a**) Resistance vs. squared current, and (**b**) zero-biased resistance *R*_0_ and resistivity vs. temperature.

The initial step voltage was selected such that it could provide a 3% rise in the thermistor resistance. Then, it was increased with the step of 200 mV until the thermistor resistance increased nearly up to 100% during the Joule heating. The resistance and TCR were measured again after Joule heating, which showed a large improvement in the resistance and TCR negatively, as shown in Figure 5a,b. In contrast to the case before Joule heating, resistivity decreases with respect to temperature.

**Figure 6:**
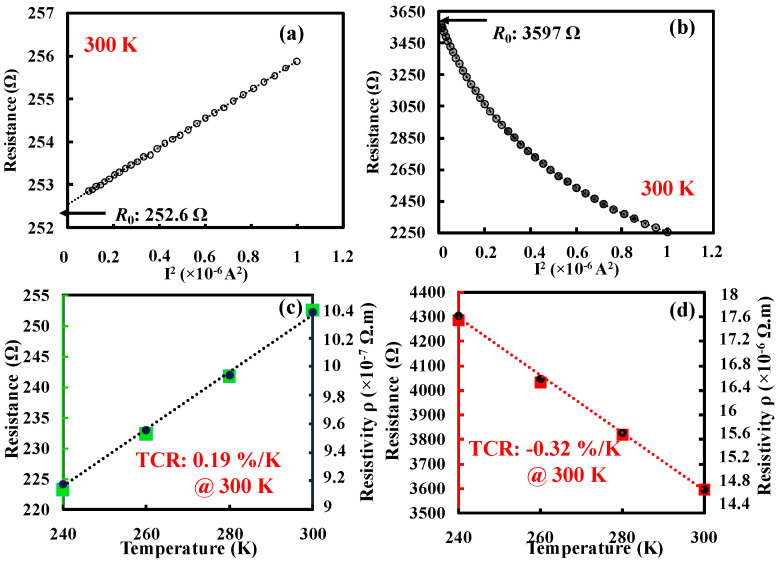
Thermistor resistance of bolometer (**a**) before and (**b**) after Joule heating. Zero-biased resistance *R*_0_ and resistivity vs. temperature (**c**) before and (**d**) after Joule heating.

Unexpectedly, the resistance became non-linear as in semiconducting material, and the TCR became negatively large. This may be caused by the impurity-induced phase change during the annealing. Based on the results attained from the suspended wire thermistor, the same procedure was applied to the bolometer with integrated heater–thermistor, whose dimensions are identical to that of a suspended wire thermistor. Since the thermistor TCR and resistance are important functions of *R*_v_, Joule heating was applied only on the thermistor. The measured resistances and TCR before and after Joule heating of the bolometer are shown in Figure 6a–d. Due to the large contribution of heater thermal conductance with the thermistor, a large heating voltage of 6.6 V was applied to the thermistor to obtain a reasonable performance improvement as in the suspended wire thermistor. Interestingly, the resistance has been increased more compared to the suspended wire thermistor, which may be due to the large applied voltage. Negatively large TCR comparable to that of the suspended wire thermistor was observed. Furthermore, annealing was carried out on two more devices with identical structure and dimensions to confirm the reproducibility. The results are analogous in that the large change in resistance and negatively large TCR are obtained after the Joule heating, as shown in Table 1. The parameter *dR*/*dI*^2^/*R*_0_^2^ in Table 1 was derived from the data presented in Figure 3a and Figure 5a for before and after Joule heating, respectively, which illustrate the correlation between resistance and the applied electrical current. The calculated slope of *R*-*I*^2^ divided by the resistance (*R*_0_) provides *dR*/*dI*^2^/*R*_0_^2^. This parameter is directly proportional to the product of the TCR, thermal resistance (*R*_t_) and the length of the suspended wire [37,52,53]. An increased *dR*/*dI*^2^/*R*_0_^2^ value, irrespective of its sign after Joule heating, signifies a noteworthy temperature rise at the center of the wire. This rise is attributed to the substantial TCR and *R*_t_, ultimately leading to a decrease in the thermal conductivity (*k*) of the Ti material and thereby enhancing the *R*_v_ characteristics.

**Figure 7:**
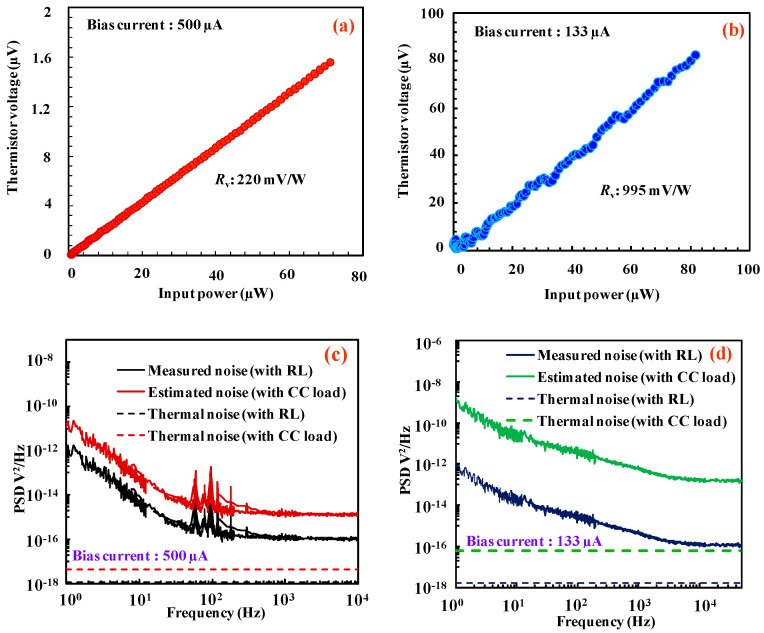
Responsivity (*R*_v_) of bolometer (**a**) before and (**b**) after Joule heating. Voltage noise (*V*_n_) power spectrum of bolometer (**c**) before and (**d**) after Joule heating.

Consequently, the important bolometer performance metrics such as *R*_v_ and NEP have been studied before and after Joule heating by applying input power to the heater and the corresponding output voltage was recorded at the thermistor. The measured results of responsivity and NEP before and after Joule heating are shown in Figure 7a–d. The *R*_v_ can be expressed by the following equation:

(1) $$R_{V}={KI}_{b}R_{0}R_{t}\alpha \;$$

where *I*_b_ is the bias current applied through the thermistor, *R*_0_ and *R*_t_ are the electrical and thermal resistances of the thermistor and α is the TCR of the thermistor. From the above equation, it is understandable that the improvement in physical constants such as *R*_0_, *R*_t_ and α could directly enhance the *R*_v_ of the bolometer. For the current bolometer, bias current of 500 µA was applied to thermistor before Joule heating which could provide a reasonable temperature rise (3% rise in resistance) at the center. 

Bias current was reduced from 500 µA to 133 µA after Joule heating to maintain the same power consumption (*P* = *I*_b_^2^ × *R*_0_, *R*_0_: Thermistor resistance at 300 K) at the thermistor. The responsivity was improved even after the bias current was reduced. The improvement in *R*_v_ can be seen in Figure 7a,b, and *R*_v_ has been increased almost 4.5 times from the condition before the Joule heating. The fluctuations in the resultant Figure 7b may be caused by the large temperature rise.

Figure 7c,d show the noise power spectrum of the fabricated bolometer before and after Joule heating, respectively. However, the improvement could not be seen in the NEP since the noise voltage increases with the thermistor resistance. A 1/*f* (flicker) noise was observed in the low-frequency region as is often seen in semiconducting materials. The thermal noise voltage of a resistive bolometer can be written as

(2) $$\bar{{{\;V}_{n}}^{2}}=4KTR_{0}$$

where *K* is the Boltzmann constant and *T* is the absolute temperature (300 K). Hence, the increase in resistance would increase the voltage noise of the device by a factor of $\sqrt{2}$. However, the large improvement in *R*_v_ could improve the NEP regardless of voltage noise since NEP is inversely proportion to *R*_v_. Since the available constant current (CC) source is noisy, a low-noise voltage source and load resistor (*R*_L_) were connected in series with the thermistor. For the fabricated titanium thermistors, a load resistor of 100 Ω was used to measure the voltage noise of the device. The effect of the load resistor was then excluded from the measured noise by subtracting the theoretical thermal noise of the load resistor from the measured noise [20]. The calculated NEPs before and after Joule heating are 1.07 µW/$\sqrt{Hz}$ and 5.01 µW/$\sqrt{Hz}$, respectively. Further analysis on material characteristics was carried out to clearly understand the phenomenon of Joule heating in Ti thermistors.

### 3.2. TEM Analysis on Ti Thermistors before and after Joule Heating

**Figure 8:**
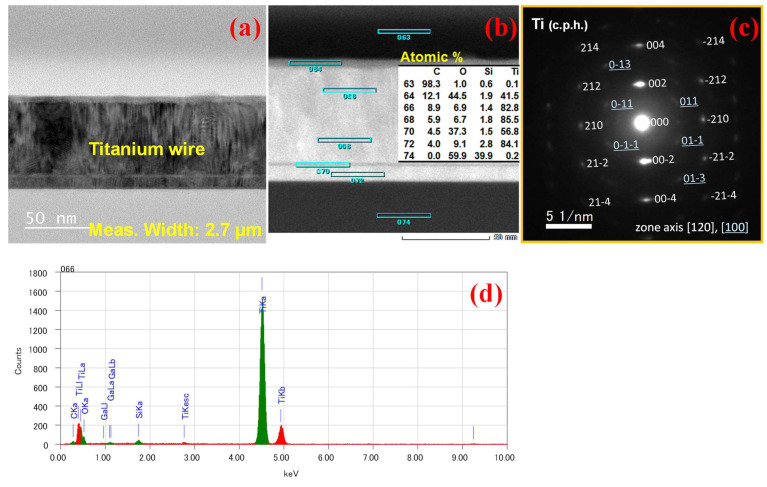
TEM/EDX analyses of a Ti thermistor before annealing. (**a**) TEM image, (**b**) HAADF-STEM image with its corresponding atomic concentration, (**c**) TED pattern with indices for Ti close-packed hexagonal (c.p.h.) crystal structure [53] and (**d**) X-ray spectrum at the position 66.

TEM analysis was performed on the Ti thermistors before Joule heating and after Joule heating to understand the result. Figure 8a–c show the TEM image of the device before Joule heating, high-angle annular dark field (HAADF)-STEM and transmission electron diffraction (TED) pattern before Joule heating, respectively. HAADF-STEM reveals that the nearly pure Ti metal with little contamination from the EB evaporation system was deposited initially. The TED pattern indicates that the film shows c-axis orientation perpendicular to the surface and includes grains with different beam directions, i.e., zone axes. The corresponding elemental mapping (EDX) is shown in Figure 8d and also confirmed the presence of almost pure Ti which has the highest atomic concentration of 85.5%.

**Figure 9:**
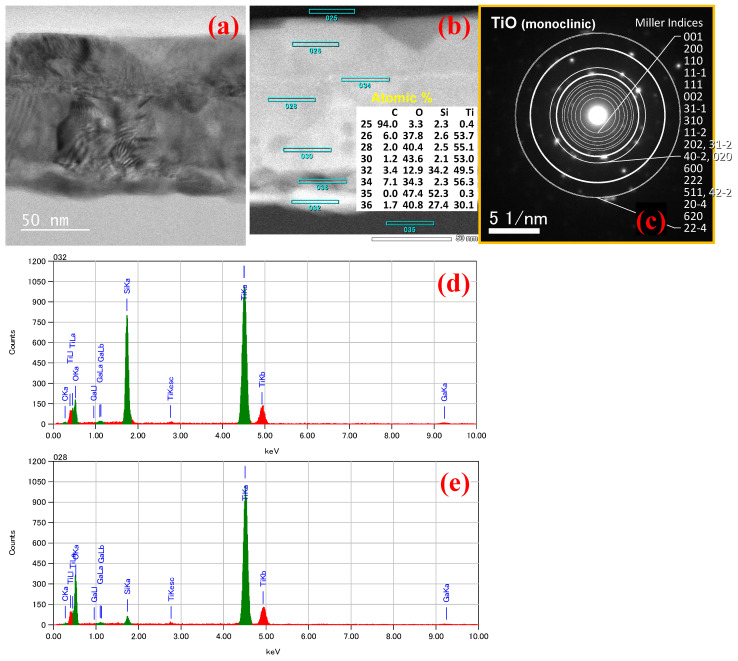
TEM/EDX analyses of a Ti thermistor after annealing. (**a**) TEM image, (**b**) HAADF-STEM image with its corresponding atomic concentration, (**c**) TED pattern with indices for polycrystalline TiO (monoclinic) [54] and X-ray spectra at positions (**d**) 32 and (**e**) 28.

A similar TEM analysis was performed on the devices after Joule heating, as represented in Figure 9. Figure 9a represents the Ti thermistor wire after Joule heating in which the SiO_2_ has reduced and the Ti has increased. Figure 9b represents the HAADF-STEM with its corresponding atomic concentration, where the formation of TiO_x_ is observed at the center. The TiO_x_ semiconducting phase may occur due to the interaction of oxygen inside the chamber with the Ti metal. Formation of TiSi_x_ was also observed at the bottom of the wire. According to the TED pattern, metallic Ti spots disappear and multiple spots that may be assigned to polycrystalline TiO appear, but some unidentified spots exist. EDX mapping represented in Figure 9d,e also confirms the formation of TiO_x_ and TiSi_x_ after Joule heating. The formation of TiO_x_ after Joule heating causes the semiconducting phase change in the material.

In the present result, conversely from the expectation, resistivity has been increased after Joule heating due to the semiconducting phase change. Formation of a TiO_x_ semiconducting phase after Joule heating could be the reason for the large negative TCR and resistance. However, in terms of the bolometer, the performance metrics directly benefit from the increased resistance and TCR. For instance, after Joule heating the responsivity improved by 4.5 times compared to the initial one.

Table 2 predicts the bolometer’s performance after Joule heating. Due to Joule heating, the resistance has increased which in turn increases the responsivity (*R*_v_). Although the absolute value of thermistor TCR has also increased slightly as a result of Joule heating, this is not included in this table since the effect is not so large. As Joule heating has directly improved the performance of the bolometer even after the semiconducting phase change, it can be concluded that Joule heating may be beneficial for improving the performances of bolometers, if there is no excess flicker noise.

## 4. Conclusions

On-chip modification of Ti thin film characteristics by Joule heating is demonstrated. Considering the importance of high resistance and TCR in improving the bolometer characteristics, the effect of the Joule heating was studied on 2.7 µm wide and 50 µm long Ti bolometers. Interestingly, a drastic increase in thermistor resistance and negatively large TCR were observed after Joule heating. Thermistor resistance was increased nearly 14 times from the initial value. The resistance has decreased after Joule heating and resultantly showed large negative TCR. *R*_v_ became 4.5 times larger although the NEP was degraded slightly. The factor *dR/dI*^2^*/R*_0_^2^ represents the increased TCR and reduced thermal conductivity (*k*) of the Ti material for better *R*_v_. TEM/EDX analysis demonstrated the formation of a TiO_x_ semiconducting phase after annealing. The Joule heating treatment is useful in increasing the bolometer output and relaxing the required input-referred noise of the readout circuit.

## Figures and Tables

**Table 1:** Variation in characteristics among three identical bolometers (*L* = 50 µm and *W* = 2.7 µm).

Device Name	Resistance (Ω)		TCR (%/K)		*dR/dI*^2^/*R*_0_^2^ (1/W)	
	Before Annealing	After Annealing	Before Annealing	After Annealing	Before Annealing	After Annealing
UR	253	3597	0.19	−0.31	52.2	−249
UL	254	3140	0.19	−0.33	52.9	−178
LR	250	4224	0.19	−0.5	47.8	−864

**Table 2:** Performance prediction due to the resistance increase by factor *K* by the Joule heating treatment.

Parameter	Factor	Remarks
Electrical resistance (*R*_e_) *	*K*	After Joule heating
Thermal resistance (*R*_t_) *	*K*	*R*_t_ ∝ *R*_e_
Bias voltage (*V*_b_)	1	
Bias current (*I*_b_), input power (*P*_in_) due to bias	1/*K*	*P*_in_ = *I*_b_^2^ × *R*_e_
Temperature rise (Δ*T*) due to bias	1	Δ*T* ∝ *P*_in_ × *R*_t_
Temperature rise by optical input (Δ*T*_opt_)	*K*	Δ*T* ∝ *P*_opt_ × *R*_t_ (*P*_opt_: const.)
Output voltage (*V*_out_)	*K*	*V*_out_ ∝ *I*_b_ × *R*_e_ × Δ*T*_opt_
Responsivity (*R*_v_)	*K*	*R*_v_ ∝ *V*_out_/*P*_opt_

* Proportional relationship between *R*_e_ and *R*_t_ is assumed.

## Data Availability

The data that support the findings of this study are available from the corresponding author (H.I.) upon reasonable request.

## References

[1] Gary S.M., Sze S.M. (2003). Fundamentals of Semiconductor Fabrication.

[2] Ziental D., Czarczynska-Goslinska B., Mlynarczyk D.T., Glowacka-Sobotta A., Stanisz B., Goslinski T., Sobotta L. (2020). Nanomaterials. Nanomaterials.

[3] Yamamoto K., Yamaguchi M., Miyamaru F., Tani M., Ikeda T., Matsushita A., Koide K., Tatsuno M., Minami Y. (2004). The Japan Society of Applied Physics Noninvasive Inspection of C-4 Explosive in Mails by Terahertz Time-Domain Spectroscopy. Jpn. J. Appl. Phys..

[4] Kawase K., Ogawa Y., Watanabe Y., Inoue H. (2003). Non-Destructive Terahertz Imaging of Illicit Drugs Using Spectral Fingerprints.

[5] Federici J.F., Schulkin B., Huang F., Gary D., Barat R., Oliveira F., Zimdars D. (2005). Semiconductor Science and Technology THz imaging and sensing for security applications—explosives, weapons and drugs. Semicond. Sci. Technol..

[6] Horowitz J.D., Thesis M.S., Chester F. (2017). Carlson Center for Imaging Science, Rochester Inst. of Tech. http://www.iupac.org/dhtml_home.html.

[7] Woolard D.L., Brown E.R., Pepper M., Kemp M. (2005). Terahertz Frequency Sensing and Imaging: A Time of Reckoning Future Applications?.

[8] Jha K.R., Singh G., Technol I.P. (2013). Terahertz planar antennas for future wireless communication: A technical review. Infrared Phys. Technol..

[9] Woodward R.M., Cole B.E., Wallace V.P., Pye R.J., Arnone D.D., Linfield E.H., Pepper M., Biol P.M. (2002). Terahertz pulse imaging in reflection geometry of human skin cancer and skin tissue. Phys. Med. Biol..

[10] Nagel M., Bolivar P.H., Brucherseifer M., Kurz H., Bosserhoff A., Buttner R. (2002). Integrated THz technology for label-free genetic diagnostics. Appl. Phys. Lett..

[11] Karpowicz N., Zhong H., Zhang C., Lin K.I., Hwang J.S., Xu J., Zhang X.C. (2005). Compact continuous-wave subterahertz system for inspection applications. Appl. Phys. Lett..

[12] Yamashita M., Otani C., Matsumoto T., Midoh Y., Miura K., Nakamae K., Nikawa K., Kim S., Murakami H., Tonouchi M. (2011). THz emission characteristics from p/n junctions with metal lines under non-bias conditions for LSI failure analysis. Opt. Express.

[13] Kan W.-C., Lee W.-S., Cheung W.-H., Wallace V.P., Pickwell-MacPherson E. (2010). Terahertz pulsed imaging of knee cartilage. Biomed. Opt. Express.

[14] Taylor Z.D., Singh R.S., Culjat M.O., Suen J.Y., Grundfest W.S., Lee H., Brown E.R. (2008). Reflective terahertz imaging of porcine skin burns. Opt. Lett..

[15] Schirmer M., Fujio M., Minami M., Miura J., Araki T., Yasui T. (2010). Biomedical applications of a real-time terahertz color scanner. Biomed. Opt. Express.

[16] Nemirovsky Y., Svetlitza A., Brouk I., Stolyarova S. (2013). Nanometric CMOS-SOI-NEMS Transistor for Uncooled THz Sensing. IEEE Trans. Electron Devices.

[17] Garn L.E. (1984). Fundamental noise limits of thermal detectors. J. Appl. Phys..

[18] Kruse P.W. (2001). Uncooled Thermal Imaging Arrays, Systems, and Applications.

[19] Hay K.A. (2006). Large format VOx microbolometer UFPA development at ITC. Infrared Detectors and Focal Plane Arrays VIII.

[20] Neikirk D.P., Lam W.W., Rutledge D.B. (1984). Far-infrared microbolometer detectors. Int. J. Infrared Millim. Waves.

[21] Shimizu T., Moritsu H., Yasuoka Y., Gamo K. (1995). Fabrication of Antenna-Coupled Microbolometers. Jpn. J. Appl. Phys..

[22] Son L.N., Tachiki T., Uchida T. (2013). Fabrication and Evaluation of Thin-Film Spiral-Antenna-Coupled VOx Microbolometer by Metal–Organic Decomposition. Jpn. J. Appl. Phys..

[23] Uchida T., Matsushita A., Tachiki T. (2014). High DC sensitivity of VOxbolometer thin films on Si3N4/SiO2membranes fabricated by metal–organic decomposition. Jpn. J. Appl. Phys..

[24] Banerjee A., Satoh H., Tiwari A., Apriono C., Rahardjo E.T., Hiromoto N., Inokawa H. (2017). Width dependence of platinum and titanium thermistor characteristics for application in room-temperature antenna-coupled terahertz microbolometer. Jpn. J. Appl. Phys..

[25] Banerjee A., Satoh H., Sharma Y., Hiromoto N., Inokawa H. (2018). Characterization of platinum and titanium thermistors for terahertz antenna-coupled bolometer applications. Sens. Actuators A Phys..

[26] Banerjee A., Satoh H., Elamaran D., Sharma Y., Hiromoto N., Inokawa H. (2018). Optimization of narrow width effect on titanium thermistor in uncooled antenna-coupled terahertz microbolometer. Jpn. J. Appl. Phys..

[27] Banerjee A., Satoh H., Elamaran D., Sharma Y., Hiromoto N., Inokawa H. (2019). Performance improvement of on-chip integrable terahertz microbolometer arrays using nanoscale meander titanium thermistor. J. Appl. Phys..

[28] Peter H.B., Nagel M., Richter F., Brucherseifer M., Kurz H., Bosserhoff A., Büttner R. (2003). Label–free THz sensing of genetic sequences: Towards ‘THz biochips’. Philos. Trans. R. Soc. A Math. Phys. Eng. Sci..

[29] Ferguson B., Zhang X.C. (2002). Materials for terahertz science and technology. Nature Mater..

[30] Tonouchi M. (2007). Cutting-edge terahertz technology. Nat. Photon..

[31] Williams B.S. (2007). Terahertz quantum-cascade lasers. Nat. Photon..

[32] Watts C.M., Shrekenhamer D., Montoya J., Lipworth G., Hunt J., Sleasman T., Krishna S., Smith D.R., Padilla W.J. (2014). Terahertz compressive imaging with metamaterial spatial light modulators. Nat. Photon..

[33] Han D.P., Fujiki R., Takahashi R., Ueshima Y., Ueda S., Lu W., Iwaya M., Takeuchi T., Kamiyama S., Akasaki I. (2021). n-type GaN surface etched green light-emitting diode to reduce non-radiative recombination centers. Appl. Phys. Lett..

[34] But D.B., Drexler C., Sakhno M.V., Dyakonova N., Drachenko O., Sizov F.F., Gutin A., Ganichev S.D., Knap W. (2014). Nonlinear photoresponse of field effect transistors terahertz detectors at high irradiation intensities. J. Appl. Phys..

[35] Yang X., Vorobiev A., Generalov A., Andersson M.A., Stake J. (2017). A flexible graphene terahertz detector. Appl. Phys. Lett..

[36] Huang X., Leng T., Zhu M., Zhang X., Chen J.C., Chang K.H., Aqeeli M., Geim A.K., Novoselov K.S., Hu Z. (2015). Highly Flexible and Conductive Printed Graphene for Wireless Wearable Communications Applications. Sci. Rep..

[37] Tiwari A., Satoh H., Aoki M., Takeda M., Hiromoto N., Inokawa H. (2015). Fabrication and analytical modeling of integrated heater and thermistor for antenna-coupled bolometers. Sens. Actuators A Phys..

[38] Elamaran D., Suzuki Y., Satoh H., Banerjee A., Hiromoto N., Inokawa H. (2020). Performance Comparison of SOI-Based Temperature Sensors for Room-Temperature Terahertz Antenna-Coupled Bolometers: MOSFET, PN Junction Diode and Resistor. Micromachines.

[39] Banerjee A., Vajandar S., Basu T. (2020). Prospects in Medical Applications of Terahertz Waves. Terahertz Biomedical and Healthcare Technologies.

[40] Samanta D., Karthikeyan M., Banerjee A., Inokawa H. (2021). Tunable graphene nanopatch antenna design for on-chip integrated terahertz detector arrays with potential application in cancer imaging. Nanomedicine.

[41] Kašalynas I., Venckevičius R., Minkevičius L., Sešek A., Wahaia F., Tamošiūnas V., Voisiat B., Seliuta D., Valušis G., Švigelj A. (2016). Spectroscopic Terahertz Imaging at Room Temperature Employing Microbolometer Terahertz Sensors and Its Application to the Study of Carcinoma Tissues. Sensors.

[42] Fukasawa R. (2015). Terahertz Imaging: Widespread Industrial Application in Non-destructive Inspection and Chemical Analysis. IEEE Transactions on Terahertz Science and Technology.

[43] Kim R.H.J., Liu Z., Huang C., Park J.M., Haeuser S.J., Song Z., Yan Y., Yao Y., Luo L., Wang J. (2022). Terahertz Nanoimaging of Perovskite Solar Cell Materials. ACS Photon..

[44] Hempel H., Savenjie T.J., Stolterfoht M., Neu J., Failla M., Paingad V.C., Kužel P., Heilweil E.J., Spies J.A., Schleuning M. (2022). Predicting Solar Cell Performance from Terahertz and Microwave Spectroscopy. Adv. Energy Mater..

[45] Nakanishi H., Ito A., Takayama K., Kawayama I., Murakami H., Tonouchi M. (2015). Comparison between laser terahertz emission microscope and conventional methods for analysis of polycrystalline silicon solar cell. AIP Adv..

[46] Yang H.U., Raschke M.B. (2016). Resonant optical gradient force interaction for nano-imaging and -spectroscopy. New J. Phys..

[47] Murakami H., Serita K., Maekawa Y., Fujiwara S., Matsuda E., Kim S., Kawayama I., Tonouchi M. (2014). Scanning laser THz imaging system. J. Phys. D Appl. Phys..

[48] Khan S., Acharyya A., Inokawa H., Satoh H., Biswas A., Dhar R.S., Banerjee A., Seteikin A.Y. (2023). Terahertz Radiation from High Electron Mobility Avalanche Transit Time Sources Prospective for Biomedical Spectroscopy. Photonics.

[49] Carranza I.E. (2015). Metamaterial Based CMOS Terahertz Focal Plane Array. Ph.D. Thesis.

[50] Cheng Y.L., Wei B.J., Shih F.H., Wang Y.L. (2012). Stability and Reliability of Ti/TiN as a Thin Film Resistor. ECS J. Solid State Sci. Technol..

[51] Tanaka A., Matsumoto S., Tsukamoto N., Itoh S., Chiba K., Endoh T., Nakazato A., Okuyama K., Kumazawa Y., Hijikawa M. (1996). Infrared focal plane array incorporating silicon IC process compatible bolometer. IEEE Trans. Electron Devices.

[52] Zhang S., Yang Y., Sadeghipour S.M., Asheghi M. Thermal Characterization of the 144 nm GMR Layer Using Microfabricated Suspended Structures. Proceedings of the ASME 2003 Heat Transfer Summer Conference.

[53] Liu W., Asheghi M. (2005). Thermal conduction in ultrathin pure and doped single-crystal silicon layers at high temperatures. J. Appl. Phys..

[54] Watanabé D., Castles J.R., Jostsons A., Malin A.S. (1967). The ordered structure of TiO. Acta Crystallogr..

